# Spinal Cord Diffuse Midline Glioma With Histone H3 K27M Mutation in a Pediatric Patient

**DOI:** 10.3389/fsurg.2021.616334

**Published:** 2021-06-17

**Authors:** Ran Cheng, Da-Peng Li, Nan Zhang, Ji-Yin Zhang, Di Zhang, Ting-Ting Liu, Jun Yang, Ming Ge

**Affiliations:** ^1^Department of Emergency Surgery, National Center for Children's Health, Beijing Children's Hospital, Capital Medical University, Beijing, China; ^2^Department of Neurosurgery, National Center for Children's Health, Beijing Children's Hospital, Capital Medical University, Beijing, China; ^3^Department of Pathology, National Center for Children's Health, Beijing Children's Hospital, Capital Medical University, Beijing, China; ^4^Department of Otolaryngology, National Center for Children's Health, Beijing Children's Hospital, Capital Medical University, Beijing, China; ^5^Department of Neurosurgery, Peking University Third Hospital, Beijing, China

**Keywords:** diffuse midline gliomas, H3K27 mutation, spinal cord tumors, pediatric neurosurgery, pathology

## Abstract

**Background:** Diffuse midline glioma (DMG) with histone H3 K27M mutation is a recently identified entity documented in the 2016 World Health Organization (WHO) Classification of Tumors of the Central Nervous System. Spinal cord DMGs with H3 K27M-mutant are commonly reported in adults. Herein, we reported a pediatric patient with spinal cord H3 K27M-mutant DMG.

**Case Report:** A 7-year-old girl with 1-month history of neck pain and 3-week history of progressive weakness in the right hand was presented. Spinal magnetic resonance imaging showed an intramedullary lesion with slight enhancement at the C2-7 levels. With intraoperative neuroelectrophysiological monitoring, the lesion was subtotally resected. Histopathological examination revealed a DMG with histone H3 K27M mutation corresponding to WHO grade IV. Postoperatively, the neck pain was relieved, and the upper-extremity weakness remained unchanged. Oral temozolomide was administrated for 7 months, and radiotherapy was performed for 22 courses. After an 18-month follow-up, no tumor recurrence was noted.

**Conclusion:** Spinal cord H3 K27M-mutant DMGs are extremely rare in pediatric patients. Preoperative differential diagnosis is challenging, and surgical resection with postoperative chemoradiotherapy may be an effective treatment.

## Introduction

H3 K27M-mutant Diffuse midline glioma (DMG) has been recognized as a distinct category in the 2016 update of the World Health Organization (WHO) Classification of Tumors of the Central Nervous System (1). This entity usually occurs in the midline of the central nervous system, and it is characterized by a specific site mutation of H3 K27M. Existing evidence indicates spinal cord H3 K27M-mutant DMGs are highly malignant tumors corresponding to WHO grade IV; nevertheless, clinical manifestations, imaging features, and treatment have not yet been well-elucidated. At present, there are few reports on the diagnosis and treatment of spinal cord H3 K27M-mutant DMGs in children. Herein, we reported a pediatric patient with spinal cord H3 K27M-mutant DMG, and relevant literature was reviewed.

## Case Report

**Figure 1:**
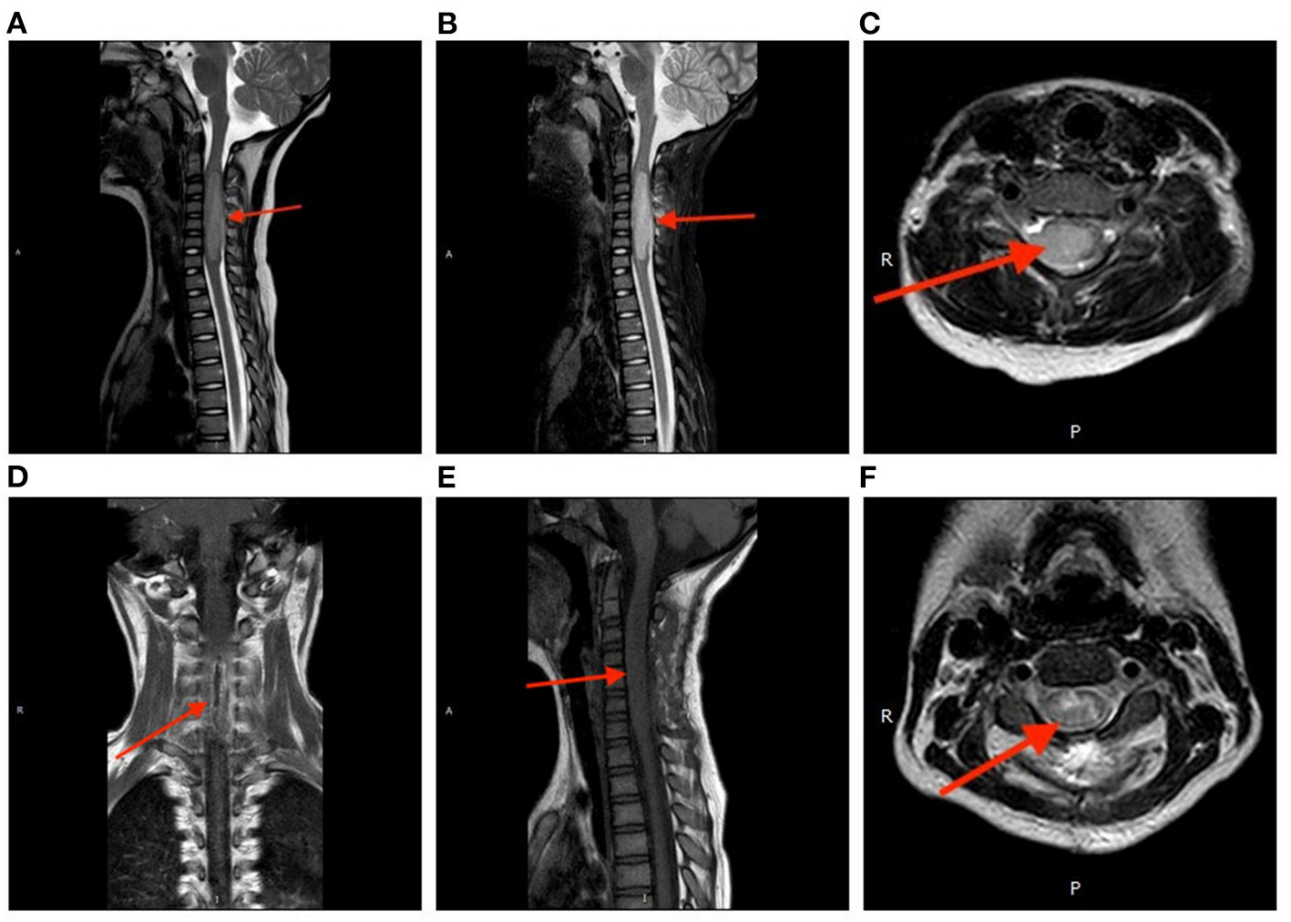
Perioperative magnetic resonance imaging. The tumor showed isointensity on T1-weighted imaging **(A)** and slightly hyperintensity on T2-weighted imaging **(B)**. After administration of contrast medium, the lesion was heterogeneously enhanced **(C)**. Postoperative spinal MRI confirmed a subtotal resection of the tumor **(D)** coronal, **(E)** sagittal, **(F)** axial.

A 7-year-old girl with a 1-month history of neck pain and a 3-week history of progressive weakness in the right hand was presented to us. Physical examination showed a decreased muscle strength (Grade 1/5 according to the Medical Research Council scale) in the right upper extremity, and the left and anterior movement of the neck was limited. The muscular tone was normal, and the Hoffmann's sign was negative bilaterally. Spinal magnetic resonance imaging (MRI) revealed an intramedullary lesion at the C2-7 levels. The lesion showed isointensity on T1-weighted imaging and slightly hyperintensity on T2-weighted imaging; after administration of contrast medium, the lesion was heterogeneously enhanced (Figures 1A–C). A diagnosis of spinal cord glioma was suspected. The preoperative ADL (Activity of Daily Living scale) score was 65.

Microsurgical resection of the intramedullary lesion was performed with the assistance of intraoperative neuroelectrophysiological monitoring. Intraoperatively, the lesion was soft in nature and pink in color with abundant blood supply. The spinal cord was severely compressed and deformed. Eventually, a subtotal resection (~80% of the mass) was achieved. Postoperatively, the neck pain was relieved, while the muscle strength of the right upper extremity remained unchanged (Grade 1/5); the muscle strength of the right lower extremity was Grade 2/5. The postoperative ADL score was 25 (Table 1).

**Table 1:** Activity of daily living scale.

**Item**	**Complete independent**	**Need some help**	**Needs a lot of help**	**Complete dependent**	**Admission**	**Discharge**	**Follow-up**
Eating	10	5	0	–	5	5	5
Washing	5	0	–	–	0	0	3
Modification	5	0	–	–	0	0	2
Dressing	10	5	0	–	5	0	2
Stool control	10	5	0	–	10	10	10
Urine control	10	5	0	–	10	10	10
Going to the toilet	10	5	0	–	5	0	5
Bed chair movement	15	10	5	0	5	0	10
Walking on the ground	15	10	5	0	15	0	15
Up and down stairs	10	5	0	–	10	0	10
					65	25	72

**Figure 2:**
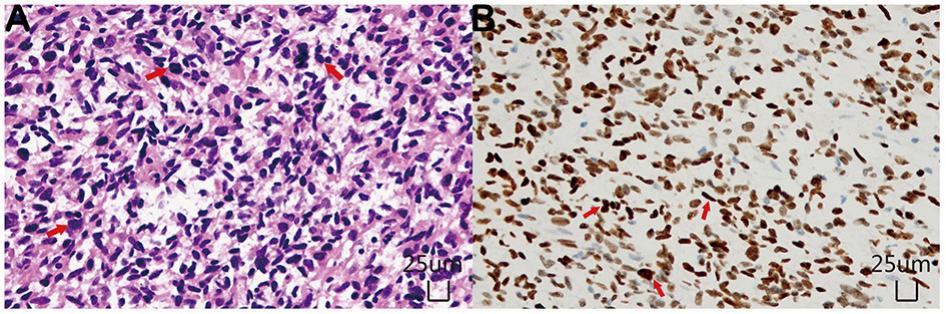
Histopathological examinations. **(A)** Hematoxylin and Eosin staining (× 200) showed diffusely distributed tumor cells with unobvious atypia and no karyokinesis. **(B)** Immunohistochemical staining (× 200) demonstrated that the tumor cells were positive for H3K27M mutant protein, and the positive site was located in the nucleus.

Postoperative spinal MRI confirmed a subtotal resection of the tumor (Figures 1D–F). Pathological examination revealed that the density of tumor cells was moderate, the nucleus was irregular and slightly to moderately heteromorphic, and no karyokinesis was observed (Figure 2A). Immunohistochemistry showed the tumor harbored an H3 K27M mutation (Figure 2B). The Ki-67 proliferation index was about 30%. These characteristics conformed to a diagnosis of spinal cord H3 K27M-mutant DMG (WHO Grade IV).

Postoperatively, the girl was treated with concurrent chemoradiotherapy. Oral temozolomide was administered for 7-months (67 mg/day for 42 days; thereafter, 150 mg/day for 5 days during each 28-day cycle). In addition, 22 courses of radiotherapy (local radiotherapy: three-dimensional conformal radiotherapy) with a total dose of 45 Gy were delivered within 5 weeks. After chemoradiotherapy, the muscle strength of right upper limb was improved, and the residual tumor was smaller than before. After an 18-month follow-up, the child's tumor did not progress, and the child's condition was stable and recovered well.

## Literature Review

Pediatric cases of spinal cord DMG with H3 K27M-mutant were retrieved from the Pubmed database. The clinical and radiological profiles were summarized in Table 2.

**Table 2:** Literature review.

**Case**	**Literature source**	**Gender (m/f)**	**Onset age (years)**	**Site of onset**	**Clinical presentation**	**Neuroimaging by MRI**	**Treatment**	**Prognosis**
1	Kumar et al. (2)	1/0	4	Cervical thoracic segment	Progressive walking difficulty 3 weeks	There were abnormally dilated and irregular areas of enhancement in the cervical and thoracic spinal cord	Symptomatic Treatment, Hormone Surgeries, Chest Core Biopsy, Plasma Exchange	Died within 4 months of the diagnosis
2	Okuda et al. (3)	1/0	10	Cervical spinal cord	Progressive weakness of right hand	Partial enhancement of intramedullary spinal cord tumor at C3-7 level	Surgery, chemoradiation, targeted therapy	Survival
3	Jung et al. (4)	1/0	13	Cervical spinal cord	NM	A well-defined and slightly hyperintense T2 signal intensity mass at cervical spinal cord, diffuse irregular rim enhancement in the tumor	NM	NM

*NM, Not mentioned*.

## Discussion

H3 K27M-mutant DMG is a new type in the fourth revision of WHO classification of central nervous system tumors. This entity represents an invasive midline high-grade glioma differentiated from astrocytes with a histone H3 K27M mutation. It predominantly occurs in the midline structure of the central nervous system, such as the brainstem, thalamus, spinal cord, the third ventricle, hypothalamus, pineal gland, and cerebellum (1–5). H3 K27M-mutant DMGs in pediatric patients are commonly located in the pons, and those arising in the spinal cord are extremely rare. There are few cases reported in the existing literature (6–8). The cervical region is the most frequently involved area of the spinal cord (9).

Its clinical manifestations are non-specific, and they vary according to the involved location of the spinal cord and the tumor growth. Local pain is the most common symptom that may occur prior to the onset of neurological deficits (10). Common neurological symptoms in children include limb weakness, abnormal gait, and sphincter dysfunctions. In the current case, the girl presented with neck pain and limb weakness, which indicates a possibility of the presence of an intramedullary tumor. It is worth noting that the symptoms in this child progressed rapidly, suggesting that the tumor was highly malignant.

Preoperative diagnosis of spinal cord H3 K27M-mutant DMG is challenging due to the absence of specific MRI characteristics. The tumor appears hypo- to is intensity on T1-weighted imaging and hyperintensity on T2-weighted imaging. Additionally, the boundaries of the tumor are ill-defined, and mild peritumoral edema may be notable. These radiological features are consistent with a high-grade glioma (4, 11, 12). Some scholars summarized that H3 K27M-mutant DMGs occurring in the cervical spinal cord usually showed uniform enhancement (13). However, in our case, the contrast enhancement of the tumor was heterogeneous, and the peritumoral spinal meninges was also thickened and enhanced. These features are different from those reported in the literature but similar to the radiological characteristics of spinal cord glioblastomas. Differential diagnoses of spinal cord H3 K27M-mutant DMGs should include ependymomas, astrocytomas, and hemangioblastomas.

H3 K27M-mutant DMGs have a broad histomorphological spectrum, varying from WHO Grade II diffuse astrocytomas to WHO Grade IV glioblastomas; in some cases, multiple forms may be concurrent in different tumor areas (14). According to the literature, the H3 K27M mutation can also be found in oligodendrocytes, giant tumor cells, epithelioid cells, rhabdomyoid tumor cells, primitive neuroectodermal tumor-like areas, pilomyxoid astrocytomas, ependymomas, and pleomorphic xanthoma astrocytomas (5). However, the prognostic relevance of the H3 K27M mutation remains unclear (15). According to the WHO classification, tumors with the H3 K27M mutation are classified into the Grade IV category, indicating a worse prognosis than the non-mutant counterparts. In our case, the histological morphology conformed to the WHO grade II diffuse astrocytoma.

Immunohistochemically, H3 K27M-mutant DMGs are usually diffusely positive to GFAP, Olig-2, and S-100 proteins. Moreover, p53 is expressed in ~50% of tumors, and expression of ATRX is absent in 15% of tumors (16–18). In this case, the spinal cord H3 K27M-mutant DMG showed diffuse immunoreactivity to Olig2, GFAP, and S-100, while the expression of ATRX was absent. The Ki-67 index was high, indicating an active proliferation. For tumors of glial origin occurring in the midline of the central nervous system in children, immunohistochemistry should be listed in the routine tests to label the H3 K27M status. When necessary, it can be further verified by molecular pathology (e.g., polymerase chain reaction or next-generation sequencing).

To date, there have been no available guidelines for the treatment of H3 K27M-mutant DMGs. Due to the infiltration into the spinal cord parenchyma, complete surgical resection of the tumor is infeasible (19–21). Adjuvant chemoradiotherapy may be helpful for prolonging progression-free survival. In the present case, ~80% of the tumor volume was removed piece by piece. The child was treated with synchronous radiotherapy and chemotherapy immediately after the surgery. A total of 22 courses of radiotherapy and six cycles of temozolomide were administrated. The disease remained stable after an 18-month follow-up. However, the efficacy of this chemoradiotherapy regimen still needs further validation in a much larger cohort.

In conclusion, we have reported on an extremely rare case of spinal cord H3 K27M-mutant DMG in a pediatric patient. After surgical resection and postoperative concurrent radio- and chemo-therapy, the prognosis is tentatively favorable.

## Data Availability Statement

The original contributions presented in the study are included in the article, further inquiries can be directed to the corresponding author/s.

## Ethics Statement

The studies involving human participants were reviewed and approved by ethics committee of Beijing Children's Hospital, Capital Medical University, National Center for Children's Health. Written informed consent to participate in this study was provided by the participants' legal guardian/next of kin. Written informed consent was obtained from the individual(s), and minor(s)' legal guardian/next of kin, for the publication of any potentially identifiable images or data included in this article.

## Author Contributions

RC and MG conceived the idea, conceptualized the study, and drafted the manuscript. D-PL and DZ collected the data. J-YZ, NZ, and JY analyzed the data. T-TL and JY reviewed the manuscript. All authors read and approved the final draft.

## Conflict of Interest

The authors declare that the research was conducted in the absence of any commercial or financial relationships that could be construed as a potential conflict of interest.
